# Stuck in the moment: cognitive inflexibility in preschoolers following an extended time period

**DOI:** 10.3389/fpsyg.2013.00959

**Published:** 2013-12-24

**Authors:** Carolina Garcia, Anthony Steven Dick

**Affiliations:** Department of Psychology, Florida International UniversityMiami, FL, USA

**Keywords:** cognitive flexibility, executive function, problem solving, Dimensional Change Card Sort, event binding

## Abstract

Preschoolers display surprising inflexibility in problem solving, but seem to approach new challenges with a fresh slate. We provide evidence that while the former is true the latter is not. Here, we examined whether brief exposure to stimuli can influence children’s problem solving following several weeks after first exposure to the stimuli. We administered a common executive function task, the Dimensional Change Card Sort, which requires children to sort picture cards by one dimension (e.g., color) and then switch to sort the same cards by a conflicting dimension (e.g., shape). After a week or after a month delay, we administered the second rule again. We found that 70% of preschoolers continued to sort by the initial sorting rule, even after a month delay, and even though they are explicitly told what to do. We discuss implications for theories of executive function development, and for classroom learning.

## INTRODUCTION

Young children appear to pick up new knowledge with ease, but they display equally surprising inflexibility in problem solving situations. No better evidence for this exists than observing children’s performance on a now-classic task, the Dimensional Change Card Sort (DCCS; Frye et al., 1995; Zelazo et al., 2003; Zelazo, 2006). In this task, children sort picture cards first by one dimension (e.g., color), and then by a second conflicting dimension (e.g., shape). Even though they are told exactly what to do (e.g., “Put the blue ones here and the yellow ones there”), when the rule changes (e.g., “Put the dogs here and the boats there”), younger preschoolers perseverate and sort by the initial rule. What remains unclear is whether this is a transient effect, or whether preschoolers who encounter conditions of conflict in a problem solving situation will carry with them the effects of that experience to the same problem solving situation if it occurs again in the more distant future.

Such an investigation must begin with an examination of why young children have difficulty with such an easy task. Several authors have focused on the processes that are called upon to overcome interference from the initial task set in the immediate context. For example, Frye et al. (1995) and Zelazo et al. (1996) proposed a Cognitive Complexity and Control (CCC) theory suggesting that in such task-switching situations children must organize the conflicting task rules into a hierarchical structure and apply that structure to determine which is the appropriate rule to use in the present context. This places a demand on working memory (Halford et al., 1998; Zelazo et al., 2003). Others suggest that, when the rule changes, children must inhibit attention to features of the object that were previously relevant in the immediate past, but are now irrelevant after the rule switch (Kirkham et al., 2003; Diamond and Kirkham, 2005; Diamond et al., 2005). This is proposed to require mature inhibitory control over the immediate context.

These theories, however, remain agnostic about how processes of working memory and inhibition interface with long term memory of prior experiences with the objects under study. In the laboratory setting this is less relevant because the stimuli used to study task-switching are often novel for the children. However, in everyday problem solving situations encountered in school and home learning settings, because it is cost-prohibitive to regularly replace the teaching tools, preschoolers often encounter objects repeatedly over the course of the school year. For example, preschool children might learn to categorize blocks or toys by their shape when they are learning shape categories, and they might then move on to learn to categorize those same objects by color. The theoretical accounts just described suggest that, in order to shift to categorize the objects by color, children would have to recruit additional cognitive resources (whether working memory or inhibitory control) to consider the same objects under a different category. However, these theories make no predictions about whether the children would have to recruit these additional resources if they encountered the objects several weeks or months after the initial experience with the objects.

Work with adults suggests that they would, and that (1) tasks carry their history with them and when task stimuli are faced again there is a re-establishment of the previous task set; (2) stimuli acquire associations with the tasks in which they occur; (3) facing the same stimuli in different tasks produces cognitive costs; (4) these effects can be detected even after long intervening time periods (Waszak et al., 2003). Additional theoretical and empirical research on memory and priming in children also suggests that children in such settings would encounter what amounts to a task-switching situation, even if the initial encounter with the objects occurred many weeks before. For example, from a theoretical perspective such a prediction would likely fit within an active-latent connectionist account of children’s performance on the DCCS. This model draws a distinction between different memory systems that are presumably engaged in tasks like the DCCS: a slow, “latent” memory system implemented in the form of connections between processing units, established over time during the pre-switch phase of the DCCS, and a fast “active” memory system implemented in the form of units capable of self-sustaining activity (Munakata, 2001; Morton and Munakata, 2002; Blackwell et al., 2009). The continued interference of the pre-switch dimension would be predicted under this model if the latent memory traces persist for long periods of time (Yerys and Munakata, 2006).

The prediction could also be made under the revised CCC theory (CCC-r; Zelazo et al., 2003; Müller et al., 2006), which specifies that negative priming of the irrelevant dimension during the pre-switch phase contributes to difficulty in the post-switch phase. Negative priming describes the disruption or slowing of a response to a stimulus that has previously been ignored (Fox, 1995; May et al., 1995). Applied to the DCCS, the theory suggests that during the pre-switch phase there is suppression or inhibition of the competing distractor (negative priming) such that when the distractor stimuli become relevant in the post-switch, the previous suppression must be overcome by application of a higher-order rule. Early theories of negative priming suggested that inhibition of the competing distractor was a transient effect (Tipper, 1985; Neill and Westberry, 1987). However, priming effects of seemingly innocuous stimuli are known to occur after months (Sloman et al., 1988; Maylor, 1998) and even years (Mitchell, 2006), even in preschool children (Drummey and Newcombe, 1995), and subsequent research has shown that negative priming effects can also last a considerable amount of time (Tipper, 2001 for review). For example, DeSchepper and Treisman (1996) observed negative priming in a selective attention task in adults after 1 month between the presentation of the prime and the subsequent presentation of the probe. This suggests that the memory traces formed even within a single experience with an object can last at full strength across several weeks of temporal delay. If negative priming contributes to difficulty on the DCCS, it would be expected that the initial experience with the stimulus properties would still contribute to interference weeks later.

There is evidence that negative priming affects performance on the DCCS over short delays of 10 min (Müller et al., 2006), but these findings provide only initial evidence that children continue to “swim against the current” if they previously encountered a problem and failed to solve it, even if that encounter occurred in the more distant past. If the effects are found to be very long lasting, it would have implications for understanding mechanisms underlying children’s cognitive inflexibility, and it would also have implications for how parents and teachers structure problem solving situations in education settings. That is, if the same materials are used to teach conflicting concepts, such a finding would suggest that some concept learning situations can actually require additional cognitive resources to overcome interference from the prior processing episode. Further, this might be the case even if the initial experience with the event was brief, and occurred several weeks or months in the past.

To investigate this issue more thoroughly, we examined whether even brief exposure to stimuli can influence problem solving following a significant intervening time after the first exposure. We administered a second post-switch phase to the DCCS following either a week or a month delay. Based on the priming literature we have reviewed, we predicted that the initial experience would have long-term effects on children’s cognitive flexibility.

## MATERIALS AND METHODS

### PARTICIPANTS

We tested sixty-two 3–5-year-old children (*M* = 4.0 *y*; SD = 0.58 *y*) from Miami-Dade, FL, USA preschools. Half participated in one of two conditions (1 week and 1 month), and did not differ on age across conditions, *t*(60) = -0.215, *p* = 0.83. Children were bilingual and were tested in their dominant language (assessed by parent questionnaire and verified by pretest; nine children were verified only by pretest). Bilingualism has been related to improved cognitive flexibility on the DCCS (Bialystok, 1999). We restricted the study to bilingual children because the majority of children in Miami-Dade preschools are bilingual, and we wanted to maintain homogeneity of the sample on this factor. Testing took place in the preschools. To control for possible effects of context, testing took place in the same location for all phases of the task.

### GENERAL DESIGN

The design was a between subjects design with Delay (1 week and 1 month) as a single factor with two levels.

We followed Zelazo (2006), with the exception that we added a second post-switch phase following the first post-switch, after either 1 week or 1 month. We used two target cards (e.g., blue dog and yellow boat) and 10 test cards (e.g., yellow dogs and blue boats). Children were randomly assigned to sort by either color or shape first, and this was not associated with task success, Fisher’s exact *p* = 0.10.

We attached one target card to each of the trays. We explained the rules for the pre-switch phase (e.g., “All the yellow ones go here, and all the blue ones go there.”) and the child watched as the first practice trial was sorted. We then asked the child to sort a card, and we provided feedback to make sure they understood the instructions. We proceeded to the pre-switch phase and asked the children to sort the remaining eight cards and place them face down into the tray. On each trial, children were reminded of the rules and asked “Here’s a (e.g., yellow one), where does this go in the (e.g., color) game?” After eight trials, we administered the post-switch rules – e.g., “Now we are going to play a new game. We are not going to play the color game anymore. We are going to play the shape game. In the shape game, all the dogs go here, and all the boats go there.” Children then sorted eight post-switch trials.

We returned to the school after an intervening period of either 1 week or 1 month. Here we only presented one set of rules identical to those given in the post-switch phase in the initial encounter. For each trial, we repeated the rule, but no feedback was given. We administered knowledge questions after the post-switch phase (Zelazo et al., 1996).

## RESULTS

Passing was defined as sorting four or more (out of eight cards) correctly. Consistent with prior work on the DCCS (Zelazo et al., 2003), the majority of children (90%) sorted correctly either all the cards, or none of the cards. All children included in analysis passed the pre-switch phase and answered the knowledge questions correctly. We first established that the groups did not differ at the first visit. Of the 31 children in each group, 12 failed the first post-switch for the 1-week delay condition, and 17 failed the first post-switch for the 1-month delay condition. These failure rates did not differ across condition, Fisher’s exact *p* = 0.37, which indicates that the pre-post differences we report after the delay cannot be attributed to differences between groups during the first visit.

Of primary interest was whether the delay would help responding. This was tested separately for both conditions, with the null hypothesis that the passing rates for the first and second post-switch phases would be equal. The pass–fail rates for each condition are given in **Table 1**. For the week condition, 9 of 12 (75%) of children who failed the first post-switch also failed the second, McNemar *χ*^2^(31) = 1.33, *p* = 0.25, suggesting no significant difference in passing rate after the week delay. Specifically, only 25% of children benefited from the delay of 1 week. For the month condition, fewer children failed. Specifically, 11 of 17 (65%) failed both post-switch phases, McNemar *χ*^2^(31) = 4.17, *p* = 0.04, which shows a significant benefit for the month delay. Thus, 35% of children passed the second post-switch after failing the first post-switch. However, the result must be interpreted within the context that only a minority of children in either condition benefited from the delay. In fact, a large percentage of children (70% across conditions) who failed the first post-switch also failed the second after a considerable intervening time period.

**Table 1:** Pass–fail rates for the first and second post-switch phases for each condition on the Dimensional Change Card Sort task.

		1 Week Delay	
		Post-switch Time 2	
		Pass	Fail
Post-switch	Pass	19	0
Time 1	Fail	3	9
	Total	22	9

		1 Week Delay	
		Post-switch Time 2	
		Pass	Fail

Post-switch	Pass	14	0
Time 1	Fail	6	11
	Total	20	11

## DISCUSSION

We administered the DCCS with a second post-switch following either a week or a month delay. We showed that while a long delay of 1 month was sufficient to facilitate shifting to the novel dimension on the DCCS, even with this considerable intervening time period, 65% of children still failed to pass the task. While it is remarkable to observe young children’s difficulty with the standard DCCS, our results extend this well-known finding to demonstrate that even brief exposure to simple stimuli can have a marked effect on children’s success in simple problem solving situations many weeks later. As we discuss, these findings have implications for theories of developing cognitive flexibility (Garon et al., 2008; Cragg and Chevalier, 2012) and for problem solving situations in educational settings.

Theoretically, two models proposed to explain performance on the DCCS can be affected by our findings because they propose processes that could potentially change, in terms of their influence on performance, over the delay period. The first are models that emphasize the role of priming and negative priming in developing cognitive flexibility (Allport and Wylie, 2000; Zelazo et al., 2003; Müller et al., 2006; Chevalier and Blaye, 2008). In particular, there is growing evidence that negative priming contributes to children’s difficulty on the DCCS (Zelazo et al., 2003; Müller et al., 2006) and on similar set-shifting tasks (Chevalier and Blaye, 2008; Dick, 2012). Further, these negative priming effects can be detected after only one or two conflicting stimulus presentations, and persist over a 10-min intervening time period (Müller et al., 2006; Experiment 4). Work with adults suggests that negative priming is not transient and can actually persist over a considerable time period (DeSchepper and Treisman, 1996), but until now this has not been shown in children. Our results connect well with the adult data in this respect, showing that negative priming of the irrelevant values may still influence the problem solving situation after several weeks (Tipper, 2001). However, the possibility that our results reflect the effects of prior negative priming would have to be confirmed, as it is possible that the findings could also be explained by interference from the previously relevant stimulus values (Kirkham et al., 2003; Chevalier and Blaye, 2008; Dick, 2012).

A second model of DCCS performance, the “active-latent” model, can also incorporate the findings we present here. As we reviewed in the Introduction, applied to the DCCS, the active-latent model proposes that flexible behavior is understood in terms of the relative strengths of “active” and “latent” memory traces (Munakata, 2001; Morton and Munakata, 2002; Blackwell et al., 2009). These memory traces are proposed to be graded in terms of the strength of the representation, and further are established over time during the pre-switch phase of the DCCS. Applied to this model, our data suggest that the latent memory trace is quite resilient in the face of considerable exposure to the stimulus values (e.g., yellow and blue) in other settings. In other words, encountering the stimulus values in another setting does not appear to “break the bond” between those particular values and the values with which they are associated during the pre-switch phase of the DCCS (e.g., boats and dogs).

The findings also fit well with models proposed for adults in task-switching situations, which suggest that tasks carry their history, and can elicit switch costs when the stimuli from the previous task are encountered in a different task (Waszak et al., 2003). The robust influence, even after long delays, of prior experience with the specific stimulus may be attributed to the retrieval of “event-bindings” comprised of the object-task-action feature associations of the initial experience (Allport and Wylie, 2000; Zmigrod and Hommel, 2013). Research shows that even repeating parts of a previous feature combination can lead to the retrieval of all components of that combination (Kahneman et al., 1992; Hommel, 2004 for review). For example, functional magnetic resonance imaging (fMRI) studies have shown that repeating a particular stimulus feature reactivates areas of the brain involved in both the representation of that feature and the representation of features that co-occurred with that feature. Thus, Keizer et al. (2008) showed that, if presented after a subject perceives a face moving across the screen in a particular direction, seeing a house move in that same direction will activate areas of cortex sensitive to features of the house and the face, even though the face is not immediately present. Kühn et al. (2011) further showed that this binding affect applies to the response as well. That is, they showed that repeating a stimulus feature leads to the neural activation of regions involved in the response, and reactivation of regions involved in a different stimulus feature that accompanied the response. Some evidence suggests that combining features (e.g., shape and location) in memory is less efficient in children (Lorsbach and Reimer, 2005; Cowan et al., 2006), but our data suggest that, even if this is the case, the binding is adequate enough to affect problem solving after a long intervening time period.

Event- or feature-binding during problem solving facilitates rapid responding to stimuli that are experienced again in the future, but this is beneficial only if the task remains the same. If the task changes, especially if it conflicts with the previous task and uses the same objects, it will lead to interference and reduced facilitation. One can readily see how this would be relevant to educational settings. If this binding is as robust as our data imply, using the same objects to emphasize a particular concept or stimulus feature would be beneficial if the concept or stimulus feature is the same, but would potentially impede learning if the concept or stimulus feature is in conflict with the previous learning episode. For example, if a teacher or parent is trying to teach a preschooler the names for shapes, it might impede learning if the same objects were previously used to teach about colors because the previous event-bindings would be recalled. Further, this could occur long after the instructor would expect the child to remember the previous experience with the objects. It remains to be determined how important this is in actual educational settings–this requires additional research. However, at a minimum our results should help educators understand the challenges that preschoolers face beyond those that are apparent in the immediate situation.

If these implications are valid, one can ask what steps can be taken to minimize the interference effects of the prior task. One option is corrective feedback, which is shown to have a significant influence on maintenance of correct responses in testing situations (Bangert-Drowns et al., 1991 for review). This is particularly important in situations in which students make mistakes, as persistence of incorrect responding is known to increase the acquisition of false knowledge (Roediger and Marsh, 2005; Butler et al., 2006). In these situations, feedback promotes the learning of correct responses (Butler et al., 2008) and predicts better performance on subsequent tests (McDaniel and Fisher, 1991; Butler and Roediger, 2008).

The effects of feedback have been specifically assessed on tasks assessing cognitive flexibility such as the DCCS. One form of feedback is labeling of the dimensions, and a number of studies have shown positive effects of labeling the relevant properties on children’s performance on the DCCS (Towse et al., 2000; Kirkham et al., 2003; Yerys and Munakata, 2006). However, both the timing of the feedback, and the nature of the labeling, affects responding (Yerys and Munakata, 2006), and some researchers have failed to find a beneficial effect of labeling on the DCCS (Müller et al., 2008). Age-related change in response to feedback is also indicated in tasks assessing cognitive flexibility. In one study, Chevalier et al. (2009) modeled the effects of feedback on an inductive task similar to the DCCS. They showed that children’s responses are affected differently by different kinds of feedback. For example, early in the task children responded well to positive feedback for the relevant color, but not negative feedback for the irrelevant colors. However, this effect changes as the task proceeds through various phases of dimensional shifts–that is, the response to feedback changes across phases of the task. Chevalier et al. (2009) also showed that age modulated feedback processing efficiency as children progressed through the task. Such findings indicate that feedback provided in situations that require cognitive flexibility can have different effects depending on the complexity of the task, how the feedback relates to the prior experiences with the stimuli, and the age of the child.

In summary, the study we report revealed a surprising finding – for preschoolers, even very brief exposure to conflicting stimuli can influence the response to those stimuli if the problem solving situation is encountered again after a long intervening time period. Evidence for the resilience of the initial representation of the stimuli should be incorporated into existing theoretical models of cognitive flexibility. Further, the results should inform future work on how to structure learning in educational settings where the available resources often require teaching sometimes conflicting concepts using the same stimuli. Our data suggest that even waiting a long time between learning opportunities is insufficient to “wash out” prior experience with the task stimuli.

## AUTHOR CONTRIBUTIONS

Anthony Steven Dick developed the study concept and design. Carolina Garcia collected the data. Anthony Steven Dick analyzed the data. Carolina Garcia and Anthony Steven Dick wrote the paper. Both authors approved the final version of the paper for submission.

## Conflict of Interest Statement

The authors declare that the research was conducted in the absence of any commercial or financial relationships that could be construed as a potential conflict of interest.
